# Prevalence and Clinical Significance of Intraventricular Conduction Disturbances in Hospitalized Children

**DOI:** 10.3390/jcdd11040129

**Published:** 2024-04-22

**Authors:** Chiara Cirillo, Emanuele Monda, Raffaella Esposito, Diego Colonna, Cristina Falcone, Federica Irrissuto, Annapaola Cirillo, Adelaide Fusco, Federica Verrillo, Gaetano Diana, Marta Rubino, Martina Caiazza, Berardo Sarubbi, Giuseppe Limongelli, Maria Giovanna Russo

**Affiliations:** 1 Inherited and Rare Cardiovascular Diseases, Department of Translational Medical Sciences, University of Campania “Luigi Vanvitelli”, Monaldi Hospital, 80131 Naples, Italy; kiaracirillo@gmail.com (C.C.);; 2 Paediatric Cardiology Unit, Department of Translational Medical Sciences, University of Campania “Luigi Vanvitelli”, Monaldi Hospital, 80131 Naples, Italy; 3 Adult Congenital Heart Diseases Unit, Monaldi Hospital, 80131 Naples, Italy

**Keywords:** interventricular conduction disturbances, paediatric cardiology, electrocardiogram

## Abstract

**Introduction:** Data on the prevalence and clinical significance of interventricular conduction disturbances (IVCDs) in children are scarce. While incomplete right bundle branch blocks (IRBBBs) seem to be the most frequent and benign findings, complete bundle blocks and fascicular blocks are often seen in children with congenital/acquired cardiac conditions. This study aims to delineate the prevalence and the diagnostic accuracy of IVCD in children admitted to a paediatric cardiology unit. **Methods:** Children admitted to the paediatric cardiology unit between January 2010 and December 2020 who had an ECG were included in the study. IVCDs were diagnosed according to standard criteria adjusted for age. **Results:** Three thousand nine hundred and ninety-three patients were enrolled. The median age was 3.1 years (IQR: 0.0–9.2 years), and 52.7% were males. IVCDs were present in 22.5% of the population: 17.4% of the population presented with IRBBBs, 4.8% with a complete right bundle branch block (CRBBB), 0.1% with a complete left bundle branch block (CLBBB), 0.2% with a left anterior fascicular block (LAFB) and 0.2% with a combination of CRBBB and LAFB. Also, 26% of children with congenital heart disease had an IVCD, and 18% of children with an IVCD had previous cardiac surgery. The overall sensitivity of IVCD in detecting a cardiac abnormality was 22.2%, with a specificity of 75.5%, a PPV of 83.1% and an NPV of 15.1%, but the values were higher for CLBBB and LAFB. **Conclusions**: IVCDs were present in one-fifth of children admitted to the cardiology unit. IRBBB was the most frequent disturbance, while CRBBB, CLBBB and fascicular blocks were much rarer, though they had a higher predictive value for cardiac abnormalities.

## 1. Introduction

**Figure 1:**
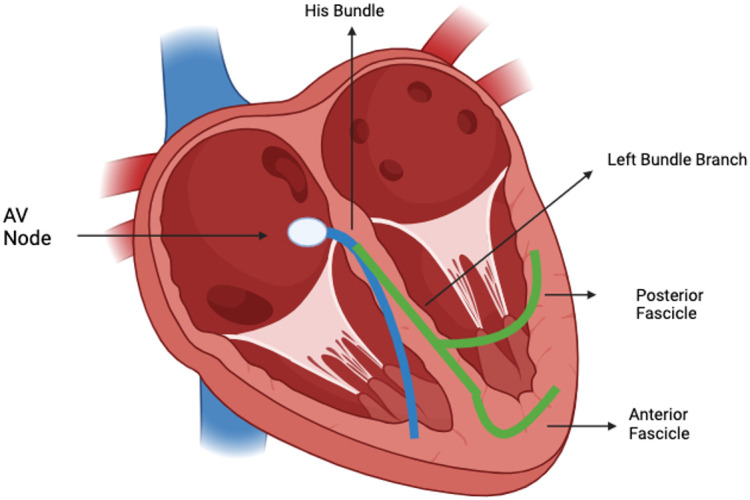
The Electrical System of the heart.

Myocardial depolarization occurs in a sequential manner via the cardiac electrical conduction system. The impulse starts in the sinus node, expands to the AV node and then, via the bundle of His, reaches the left and right ventricular branches simultaneously (Figure 1). Intraventricular conduction delays (IVCDs) occur when there is an interruption in the transmission to the ventricles, generating bundle blocks, left bundle branch blocks (LBBBs) and right bundle branch block (RBBBs). The left branch is divided into two smaller branches, the left anterior and posterior branches, and a conduction disorder involving these branches is known as a fascicular block—a left anterior fascicular block (LAFB) or a left posterior fascicular block (LPFB) [1,2].

IVCDs are easily recognizable on surface electrocardiograms (ECGs), and their prevalence and correlation with cardiac disease has been thoroughly investigated in adult populations [3,4,5].

The prevalence and clinical significance of IVCDs in children is not completely understood. While incomplete right bundle branch blocks (IRBBBs) are commonly identified in paediatric ECGs and are considered benign findings, complete right bundle branch blocks (CRBBBs), complete left bundle branch blocks (CLBBBs) and fascicular blocks are much rarer and are often associated with congenital heart disease (CHD) and/or open-heart surgery [6,7].

This study aimed to delineate the prevalence of IVCDs and their clinical significance in a large cohort of children admitted to a paediatric cardiology unit in a large tertiary centre in Italy.

## 2. Methods

The study adhered to the principles of the Declaration of Helsinki and was approved by the local institutional ethics committee. Admissions to the Paediatric Cardiology Ward (Monaldi Hospital, Naples, Italy) between January 2010 and December 2020 were retrospectively screened.

All patients hospitalized in the paediatric cardiology ward and the paediatric cardiac intensive care unit (PICU) were included if they had an electrocardiogram (ECG) recorded on the first day of admission. Exclusion criteria were age over 18, unreadable ECG traces and paced rhythm.

Age at admission, gender, reason for admission, previous cardiac surgery and clinical examination findings were recorded.

### 2.1. Electrocardiography

A 12-lead ECG was recorded with the subjects in the supine position and was analyzed by two experienced cardiologists. IVCDs were diagnosed as follows.

### 2.2. Right Bundle Branch Block (RBBB)

RBBB was defined as the presence of rsR’ or sR’ in V1 with R’ greater than r or a R’ peak duration of 60 ms or more. When the QRS duration was >0.12 s, it was defined as complete RBBB (CRBBB); when the QRS duration was <0.12 s, it was defined as incomplete RBBB (IRBBB) [8,9].

### 2.3. Left Bundle Branch Block (LBBB)

Complete LBBB (CLBBB) was defined as the presence of a prolonged QRS >0.12 s or delayed intrinsic deflection in lead V6 (>75 ms), an absent Q wave, and slurred broad R waves in leads I, aVL and V6 [8,9].

### 2.4. Left Anterior Fascicular Block (LAFB)

LAFB was defined as the presence of left axis deviation, qR morphology in aVL, lead I and rS morphology in lead II, and aVF or rS in lead 2 with an S wave deeper in lead II than in lead III and a QRS duration < 0.12 s [8,9].

### 2.5. Left Posterior Fascicular Block (LPFB)

LPFB presents as an axis between 90° and 180° (not applicable at <16 years old due to the physiologically more rightward axis in children) and an rS pattern in leads I and aVL, a qR pattern in leads III and aVF, and a QRS duration less than 0.12 s [8,9].

### 2.6. Age Adjustments

In younger children and infants, the QRS duration was adjusted by age considering that a normal QRS duration is <0.09 s up to three years of age and <0.10 s for children up to adolescence and <0.12 s for 12 years and above [10,11].

### 2.7. Statistical Analysis

Data are presented as mean values and standard deviations if normally distributed and as medians with interquartile ranges (IQRs) if skewed. Categorical data are presented as counts divided by the total number of valid/available data. To assess the diagnostic value of each IVCD, a 2 × 2 table was constructed that included true- and false-positive and true- and false-negative test results. From the 2 × 2 frequency data, the sensitivity and specificity in detecting cardiac abnormalities were determined.

Diagnostic accuracy was defined as the total number of true-positive and true-negative tests/total number of patients. Positive predictive value (PPV), negative predictive value (NVP) and predictive accuracy were calculated and presented as absolute numbers and 95% confidence intervals.

## 3. Results

The clinical records of 4015 patients were screened. In total, 3 patients were 18 or older, 10 ECGs showed paced rhythm and 9 traces were not readable. The study enrolled a total of 3993 patients. The median age was 3.1 years (IQR: 0.0–9.2 y), and 52.7% were males.

**Figure 2:**
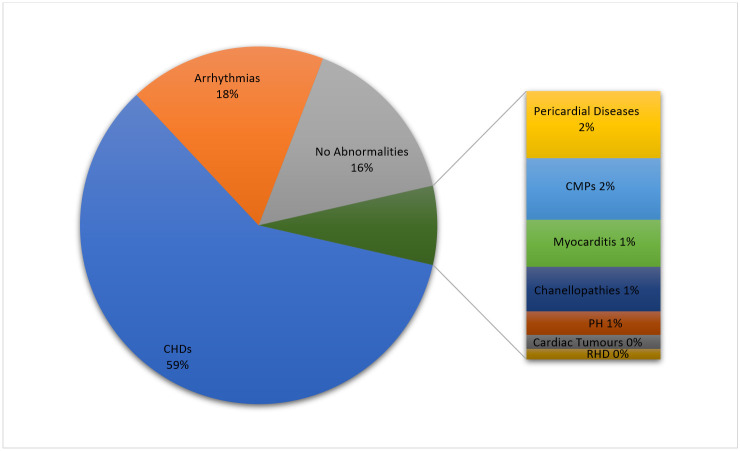
Distribution of aetiologies in the overall cohort. Abbreviations: CHDs, congenital heart diseases; CMPs, cardiomyopathies; PH, pulmonary hypertension; RDH, rheumatic heart disease.

The reasons for admission were CHD (59.33%), arrhythmia (17.8%), pericardial disease (1.63%), myocarditis (1.25%), channelopathies (1.18%), pulmonary hypertension (0.63%), cardiac tumour (0.38%), rheumatic heart disease (0.27%) and Kawasaki disease (0.27%). Only 15.52% of the admitted patients did not present any structural cardiac abnormalities at admission (Table 1, Figure 2). There were eight patients with dextrocardia, two of which cases were in the setting of situs inversus.

**Figure 3:**
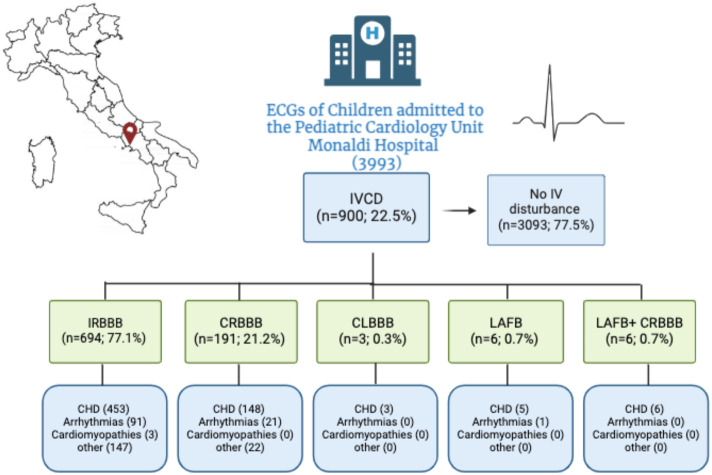
Prevalence of IVCDs in our cohort. Abbreviations: IVCD, intraventricular conduction disturbance; IV, intraventricular; IRBBB, incomplete right bundle branch block; CRBBB, complete right bundle branch block; CLBBB, complete left bundle branch block; LAFB, left anterior fascicular block; CHD, congenital heart disease.

IVCDs were present in 22.54% of the population. In total, 17.38% of the population presented with IRBBB, 4.78% with CRBBB, 0.07% with CLBBB, 0.15% with LAFB and 0.15% with a combination of CRBBB and LAFB (Figure 3).

The distribution of IVCDs among different cardiac conditions was as follows: 68.4% were seen in children with CHD, 14.7% in children with arrhythmias and channelopathies, 14.4% in children with no morpho-functional cardiac abnormalities, 1.3% in those with cardiomyopathies/myocarditis and 1.3% in those with other rarer diseases (cardiac tumours, pulmonary hypertension and Kawasaki disease).

### 3.1. Association between IVCD and Cardiac Surgery

Of the 3993 children included in the study, 242 (6%) underwent open-heart surgery prior to the index admission. Eighteen percent of the children with IVCDs had previous cardiac surgery. The frequency of previous cardiac surgery was 10% in IRBBB, 41% in CRBBB, 83.5% in LAFB and 100% in both the LBBB and LAFB+ CRBBB groups. Table 2 summarizes the previous surgical procedures for each IVCD.

### 3.2. Association between Heart Diseases and IVCD Subtypes

Table 3 and Table 4 summarize the prevalence of each IVCD across different cardiac conditions.

### 3.3. CHD

Twenty-six percent of the children with CHD had an IVCD. The most frequent IVCD among the CHD patients was IRBBB (19% of all CHD patients), followed by CRBBB (6.24%); CLBBB, LAFB and CRBBB+ LAFB were much rarer (0.12%, 0.25% and 0.25%, respectively).

IRBBB was most frequently seen with ostium secundum atrial septal defect (ASD) (26.33%), pulmonary atresia with intact septum (24.2%), pulmonary atresia with ventricular septal defect (VSD) (22.7%), coarctation of the aorta (22.05%), pulmonary valve dysplasia (21%) and ostium primum ASD (18%).

CRBBB was most frequently seen with tetralogy of Fallot (26.1%), pulmonary atresia with VSD (18.2%) and tricuspid valve dysplasia (11.7%).

All six patients with CRBBB + LAFB had CHD, five of whom had an atrioventricular septal defect.

Three children in our cohort presented with CLBBB, and they all had CHD (truncus arteriosus, great vessel transposition and aortic stenosis).

### 3.4. Arrhythmias

Among patients presenting with a rhythm disturbance, 12.7% had an IVCD. The most frequent was IRBBB, seen in 12.7% of children, followed by CBBB, seen in 2.9% of children. IRBBB was also present in five (25%) children diagnosed with Brugada syndrome and four children with long QT syndrome.

### 3.5. Diagnostic Accuracy of Paediatric IVCD

**Figure 4:**
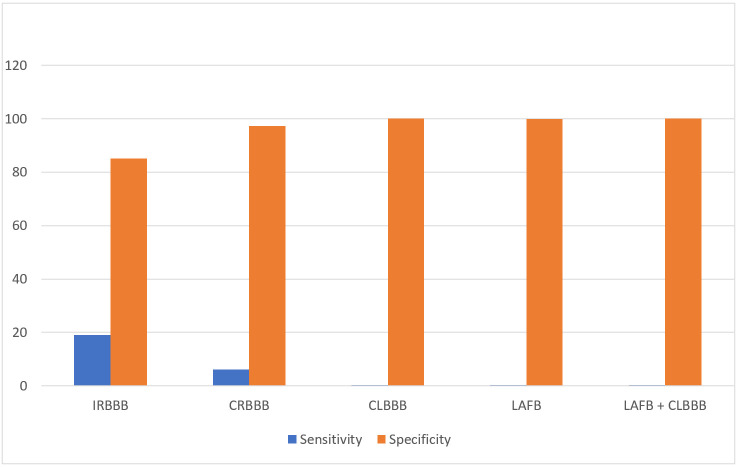
Sensitivity and specificity of IVCD.

The overall sensitivity of IVCD in detecting a cardiac abnormality was 22.2%, with a specificity of 75.48%, a PPV of 83.11%, an NPV of 15.13%, a positive likelihood ratio of 0.9, a negative likelihood ratio of 1.03, a prevalence of 84.47% and a 30.45% predictive accuracy (Figure 4).

IRBBB had a 19.1% mean sensitivity and an 85.1% specificity for CHD. With regard to CRBBB, the highest sensitivity was found for Ebstein anomaly (37.5%), followed by tetralogy of Fallot (26.1%).

CLBBB, LAFB and CRBBB + LAFB all had a specificity > 99.99% for CHD.

The sensitivity, specificity, PPV and NPV for each disease group and the sensitivity and specificity for individual diseases are reported in the Supplementary Materials (Tables S1–S5).

## 4. Discussion

This study describes the prevalence and clinical significance of IVCD in a paediatric population admitted to a paediatric cardiology unit. These data can be useful to understand the association of IVCD with paediatric cardiac conditions.

### 4.1. IRBBB and CRBBB

IVCDs are rare in children and were infrequent even in our selected population of children hospitalized for suspected/known cardiac conditions.

Overall, the most common IVCD in our cohort was IRBBB, followed by CRBBB.

Previous studies described a prevalence of IRBBB in 0.36% of children screened in a school program excluding those with CHD [12], in 3% of children with an ECG recorded in an outpatient setting [7] and in 1.2% of male highschoolers [13]. We showed a significantly higher prevalence (17.3%) compared to previous data, likely reflective of the hospitalized cohort with known or suspected heart disease.

Data on the clinical significance of IRBBBs in otherwise healthy children are scarce. IRBBB in a very young child can be considered a normal variant due to the persistence of right ventricular predominance; however, it is an unusual pattern for an older child [7].

IRBBBs can be observed in children with ASDs, and while previous data showed a lack of specificity, we reported a specificity of 85% and 82.5% for ostium secundum ASD and ostium primum ASD, respectively [14,15].

CRBBB is quite rare in children; it is reported to have a male preference and to become more frequent with age [16,17]. We reported a low prevalence of CRBBBs within our selected population, and we confirmed the known association with CHD before and especially after surgical repair. The right bundle branch is vulnerable to surgery in the proximal and distal regions, and IRBBBs and CRBBBs are described after repair of tetralogy of Fallot, atrioventricular septal defect and VSD [18,19,20].

Probst and colleagues reported a progressive cardiac conduction delay in families affected by Brugada syndrome with a *SCN5A* mutation. In our study, 25% of children with confirmed Brugada syndrome presented with IRBBBs and 5% with CRBBBs [21].

### 4.2. CLBBB and LAFB

Although CLBBB is seen in up to 2% of the adult population [22], it is very rare in children and mostly seen after open-heart surgery on the left side of the heart and rarely in cardiomyopathies or myocarditis. Our data confirm the rarity and the association in 100% of patients with previous cardiac surgery. Accuracy tests for CLBB and LAFB are reported but strongly limited by the small sample size.

LAFB and the association of LAFB + CRBBB, similarly to CLBBB, is also associated with structural heart disease, mostly with AV septal defects and coronary abnormalities, which was confirmed in our cohort [23].

## 5. Conclusions

To our knowledge, this is the first study reporting the prevalence and diagnostic accuracy of IVCD in admitted children with known/suspect cardiac conditions.

The main limitations of the study are the retrospective nature of the study and the selected population.

In conclusion, this study confirms the rarity of IVCD in children, even in those admitted for cardiac conditions, and that the presence of IVCD must warrant further investigations given its association with congenital, inherited or acquired cardiac conditions. CBBB and LAFB showed the strongest association with structural heart disease.

## Figures and Tables

**Table 1:** Clinical characteristics of the study cohort. Data are presented as mean ± SD, median (IQR) or *n* (%).

Clinical Features	Overall Cohort (*n* = 3993)
Age, years	3.1 (0.0–9.2)
Males	2103 (52.7)
Congenital heart diseases	2369 (59.33)
Patent foramen ovale	109 (2.73)
Secundum atrial septal defect	846 (21.19)
Primum atrial septal defect	11 (0.27)
Atrioventricular septal defect	77 (1.93)
Ventricular septal defect	450 (11.27)
Patent ductus arteriosus	523 (13.10)
Aortopulmonary window	10 (0.25)
Abnormal origin of coronary artery from inappropriate situs	14 (0.35)
Anomalous left coronary artery from the pulmonary artery	2 (0.05)
Anomalies of the aortic arch	61 (1.53)
Aberrant origin of the subclavian artery	9 (0.22)
Partially anomalous pulmonary venous connection	2 (0.05)
Totally anomalous pulmonary venous connection	19 (0.48)
Tricuspid valve atresia	22 (0.55)
Tricuspid valve dysplasia	17 (0.43)
Ebstein anomaly	8 (0.20)
Tricuspid stenosis	1 (0.02)
Pulmonary stenosis	183 (4.58)
Pulmonary valve dysplasia	38 (0.95)
Pulmonary atresia and intact ventricular septum	33 (0.83)
Pulmonary atresia and ventricular septal defect	22 (0.55)
Tetralogy of Fallot	157 (3.93)
Truncus arteriosus	8 (0.20)
Mitral valve abnormalities	52 (1.30)
Bicuspid aortic valve	33 (0.83)
Aortic valve stenosis	52 (1.30)
Aortic subvalvular stenosis	4 (0.10)
Coarctation of the aorta	127 (3.18)
Hypoplastic left heart syndrome	17 (0.43)
Transposition of the great arteries	64 (1.60)
Congenitally corrected transposition of the great arteries	6 (0.15)
Double outlet right ventricle	44 (1.10)
Double inlet left ventricle	11 (0.27)
Cor triatriatum	3 (0.07)
Abnormalities of atrial situs	16 (0.40)
Cardiomyopathies	65 (1.63)
Hypertrophic cardiomyopathy	34 (0.85)
Dilated cardiomyopathy	28 (0.70)
Restrictive cardiomyopathy	1 (0.02)
Left ventricular non compaction	2 (0.05)
Rheumatic heart disease	11 (0.27)
Myocarditis	50 (1.25)
Pericardial disease	71 (1.78)
Acute pericarditis	67 (1.68)
Chronic pericarditis	4 (0.10)
Kawasaki disease	8 (0.20)
Pulmonary hypertension	25 (0.63)
Cardiac tumours	15 (0.38)
Rhabdomyomas	15 (0.38)
Disorder of the cardiac rhythm	712 (17.83)
Frequent supraventricular premature complexes	48 (1.20)
Frequent ventricular premature complexes	78 (1.95)
Ventricular pre-excitation	130 (3.26)
Atrial ectopic tachycardia	130 (3.26)
Atrioventricular re-entry tachycardia	237 (5.93)
Atrioventricular node re-entry tachycardia	134 (3.36)
Atrial flutter	15 (0.38)
Atrial fibrillation	4 (0.10)
Ventricular tachycardia	7 (0.17)
Cardiac channelopathies	47 (1.18)
Long QT syndrome	27 (0.68)
Brugada syndrome	20 (0.50)
No morpho-functional abnormalities	620 (15.52)
Cardiac surgery prior to admission	242 (0.6)

**Table 2:** Cardiac repairs associated with intraventricular delay subtypes.

	TOF	CoA	VSD	ASD	AVSD	PDA	Shone Complex	PA + VSD	Arterial Switch	Glenn/Fontan	DORV	Truncus	Tot
**IRBBB**	13	16	13	6	5	0	4	3	10	4	1	0	75
**CRBBB**	36	5	8	5	2	2	0	6	2	2	4	0	72
**CLBBB**	1	0	0	0	0	0	0	0	1	0	0	2	4
**LAFB**	0	0	0	0	3	0	0	1	1	0	0	0	5
**CRBBB + LAFB**					5						1		6

*Abbreviations*: TOF, tetralogy of Fallot; CoA, aortic coarctation; ASD, atrial septal defect; VSD, ventricular septal defect; AVSD, atrioventricular septal defect; PDA, patent ductus arteriosus; PA, pulmonary atresia, DORV, double outlet right ventricle; IRBBB, incomplete right bundle branch block; CRBBB, complete right bundle branch block; LAFB, left anterior fascicular block; CLBBB, complete left bundle branch block.

**Table 3:** Clinical characteristics of the overall cohort according to the type of intraventricular conduction delay. Data are presented as mean ± SD, median (IQR) or *n* (%).

	IRBBB (*n* = 694)	Isolated CRBBB (*n* = 191)	CLBBB (*n* = 3)	Isolated LAFB (*n* = 6)	CRBBB + LAFB (*n* = 6)
Age, years	6.0 (1–11)	7.5 (4–12)	10 (8.5–17)	3.5 (0.2–9.7)	2.5 (0–11)
Males	393 (56.6)	123 (64.4)	1 (33.3)	1 (16.7)	3 (50)
Previous Cardiac Surgery	75 (10.8)	72 (37.7)	3 (100)	5 (83.5%)	6 (100)
Congenital heart diseases	453 (65.3)	148 (77.5)	3 (100)	5 (83.3)	6 (100)
Patent foramen ovale	9 (1.30)	5 (2.62)	0 (0)	0 (0)	0 (0)
Secundum atrial septal defect	223 (32.13)	43 (22.51)	0 (0)	1 (16.7)	0 (0)
Primum atrial septal defect	2 (0.29)	0 (0)	0 (0)	0 (0)	0 (0)
Atrioventricular septal defect	8 (1.15)	7 (3.66)	0 (0)	2 (33.3)	5 (83.3)
Ventricular septal defect	58 (8.36)	28 (14.66)	0 (0)	1 (16.7)	0 (0)
Patent ductus arteriosus	82 (11.82)	10 (5.24)	0 (0)	1 (16.7)	0 (0)
Aortopulmonary window	2 (0.29)	1 (0.52)	0 (0)	0 (0)	0 (0)
Abnormal origin of coronary artery from inappropriate situs	3 (0.43)	1 (0.52)	0 (0)	0 (0)	0 (0)
Anomalous left coronary artery from the pulmonary artery	0 (0)	0 (0)	0 (0)	0 (0)	0 (0)
Anomalies of the aortic arch	11 (1.58)	3 (1.57)	0 (0)	0 (0)	0 (0)
Aberrant origin of the subclavian artery	2 (0.29)	0 (0)	0 (0)	0 (0)	0 (0)
Partially anomalous pulmonary venous connection	0 (0)	1 (0.52)	0 (0)	0 (0)	0 (0)
Totally anomalous pulmonary venous connection	3 (0.43)	2 (1.05)	0 (0)	0 (0)	0 (0)
Tricuspid valve atresia	1 (0.14)	0 (0)	0 (0)	1 (16.7)	0 (0)
Tricuspid valve dysplasia	1 (0.14)	2 (1.05)	0 (0)	0 (0)	0 (0)
Ebstein anomaly	1 (0.14)	3 (1.57)	0 (0)	0 (0)	0 (0)
Tricuspid stenosis	0 (0)	1 (0.52)	0 (0)	0 (0)	0 (0)
Pulmonary stenosis	34 (4.90)	9 (4.71)	0 (0)	0 (0)	0 (0)
Pulmonary valve dysplasia	8 (1.15)	0 (0)	0 (0)	0 (0)	0 (0)
Pulmonary atresia and intact ventricular septum	8 (1.15)	0 (0)	0 (0)	0 (0)	0 (0)
Pulmonary atresia and ventricular septal defect	5 (0.72)	4 (2.09)	0 (0)	0 (0)	0 (0)
Tetralogy of Fallot	11 (1.58)	41 (21.47)	0 (0)	1 (16.7)	0 (0)
Truncus arteriosus	0 (0)	0 (0)	1 (33.3)	0 (0)	0 (0)
Mitral valve abnormalities	10 (1.44)	4 (2.09)	0 (0)	0 (0)	0 (0)
Bicuspid aortic valve	4 (0.58)	2 (1.05)	0 (0)	0 (0)	0 (0)
Aortic valve stenosis	7 (1.01)	1 (0.52)	1 (33.3)	0 (0)	0 (0)
Aortic subvalvular stenosis	1 (0.14)	0 (0)	0 (0)	0 (0)	0 (0)
Coarctation of the aorta	28 (4.03)	15 (7.85)	0 (0)	0 (0)	0 (10)
Hypoplastic left heart syndrome	2 (2.29)	1 (0.52)	0 (0)	0 (0)	0 (0)
Transposition of the great arteries	10 (1.44)	6 (3.14)	1 (33.3)	1 (16.7)	0 (0)
Congenitally corrected transposition of the great arteries	0 (0)	0 (0)	0 (0)	0 (0)	0 (0)
Double outlet right ventricle	4 (0.58)	7 (3.66)	0 (0)	0 (0)	1 (16.7)
Double inlet left ventricle	1 (0.14)	1 (0.52)	0 (0)	0 (0)	0 (0)
Cor triatriatum	0 (0)	0 (0)	0 (0)	0 (0)	0 (0)
Abnormalities of atrial situs	0 (0)	1 (0.52)	0 (0)	0 (0)	0 (0)
Cardiomyopathies	3 (0.14)	0 (0)	0 (0)	0 (0)	0 (0)
Hypertrophic cardiomyopathy	2 (0.29)	0 (0)	0 (0)	0 (0)	0 (0)
Dilated cardiomyopathy	1 (0.14)	0 (0)	0 (0)	0 (0)	0 (0)
Restrictive cardiomyopathy	0 (0)	0 (0)	0 (0)	0 (0)	0 (0)
Left ventricular non compaction	0 (0)	0 (0)	0 (0)	0 (0)	0 (0)
Rheumatic heart disease	1 (0.14)	0 (0)	0 (0)	0 (0)	0 (0)
Myocarditis	8 (1.15)	0 (0)	0 (0)	0 (0)	0 (0)
Pericardial disease	14 (2.02)	1 (0.52)	0 (0)	0 (0)	0 (0)
Acute pericarditis	13 (1.87)	1 (0.52)	0 (0)	0 (0)	0 (0)
Chronic pericarditis	1 (0.14)	0 (0)	0 (0)	0 (0)	0 (0)
Kawasaki disease	1 (0.14)	0 (0)	0 (0)	0 (0)	0 (0)
Pulmonary hypertension	3 (0.43)	1 (0.52)	0 (0)	0 (0)	0 (0)
Cardiac tumours	1 (0.14)	0 (0)	0 (0)	0 (0)	0 (0)
Rhabdomyomas	1 (0.14)	0 (0)	0 (0)	0 (0)	0 (0)
Disorder of the cardiac rhythm	91 (13.11)	21 (10.99)	0 (0)	1 (16.7)	0 (0)
Frequent supraventricular premature complexes	4 (0.58)	0 (0)	0 (0)	0 (0)	0 (0)
Frequent ventricular premature complexes	10 (1.44)	2 (1.05)	0 (0)	0 (0)	0 (0)
Ventricular pre-excitation	13 (1.87)	2 (1.05)	0 (0)	1 (16.7)	0 (0)
Atrial ectopic tachycardia	13 (1.87)	10 (5.24)	0 (0)	0 (0)	0 (0)
Atrioventricular re-entry tachycardia	29 (4.18)	6 (3.14)	0 (0)	0 (0)	0 (0)
Atrioventricular node re-entry tachycardia	26 (3.75)	6 (3.14)	0 (0)	0 (0)	0 (0)
Atrial flutter	1 (0.14)	0 (0)	0 (0)	0 (0)	0 (0)
Atrial fibrillation	0 (0)	0 (0)	0 (0)	0 (0)	0 (0)
Ventricular tachycardia	2 (2.9)	0 (0)	0 (0)	0 (0)	0 (0)
*Cardiac channelopathies*	9 (1.30)	*1 (0.52)*	*0 (0)*	*0 (0)*	*0 (0)*
Long QT syndrome	4 (0.58)	0 (0)	0 (0)	0 (0)	0 (0)
Brugada syndrome	5 (0.72)	1 (0.52)	0 (0)	0 (0)	0 (0)
No morpho-functional abnormalities	110 (15.85)	19 (9.95)	0 (0)	0 (0)	0 (0)

Abbreviations: CLBBB, complete left bundle branch block; CRBBB, complete right bundle branch block; IRBBB, incomplete right bundle branch block; LAFB, left anterior fascicular block.

**Table 4:** Association between heart diseases and intraventricular conduction delay. Data are presented as mean ± SD, median (IQR), or *n* (%).

	IRBBB (*n* = 694)	Isolated CRBBB (*n* = 191)	CLBBB (*n* = 3)	Isolated LAFB (*n* = 6)	CRBBB + LAFB (*n* = 6)	No Abnormalities
Congenital heart diseases (*n* = 2369)	453 (19.12)	148 (6.25)	3 (0.13)	5 (0.21)	6 (0.25)	1754 (74.04)
Patent foramen ovale (*n* = 109)	9 (8.26)	5 (4.59)	0 (0)	0 (0)	0 (0)	95 (87.15)
Secundum atrial septal defect (*n* = 846)	223 (26.33)	43 (5.08)	0 (0)	1 (0.12)	0 (0)	579 (68.36)
Primum atrial septal defect (*n* = 11)	2 (18.18)	0 (0)	0 (0)	0 (0)	0 (0)	9 (81.82)
Atrioventricular septal defect (*n* = 77)	8 (10.39)	7 (9.09)	0 (0)	2 (2.60)	5 (6.49)	55 (71.43)
Ventricular septal defect (*n* = 450)	58 (12.80)	28 (6.18)	0 (0)	1 (0.22)	0 (0)	363 (80.13)
Patent ductus arteriosus (*n* = 523)	82 (15.35)	10 (1.87)	0 (0)	1	0 (0)	430 (80.52)
Aortopulmonary window (*n* = 10)	2 (20)	1 (10)	0 (0)	0 (0)	0 (0)	7 (70)
Abnormal origin of coronary artery from inappropriate situs (*n* = 14)	3 (21.43)	1 (7.14)	0 (0)	0 (0)	0 (0)	10 (71.43)
Anomalous left coronary artery from the pulmonary artery (*n* = 2)	0 (0)	0 (0)	0 (0)	0 (0)	0 (0)	2 (100)
Anomalies of the aortic arch (*n* = 61)	11 (18.03)	3 (4.92)	0 (0)	0 (0)	0 (0)	47 (77.05)
Aberrant origin of the subclavian artery (*n* = 9)	2 (22.22)	0 (0)	0 (0)	0 (0)	0 (0)	7 (77.77)
Partially anomalous pulmonary venous connection (*n* = 2)	0 (0)	1 (50)	0 (0)	0 (0)	0 (0)	1 (50)
Totally anomalous pulmonary venous connection (*n* = 19)	3 (15.79)	2 (10.53)	0 (0)	0 (0)	0 (0)	14 (73.68)
Tricuspid valve atresia (*n* = 22)	1 (4.54)	0 (0)	0 (0)	1 (4.54)	0 (0)	20 (90.91)
Tricuspid valve dysplasia (*n* = 17)	1 (5.88)	2 (11.76)	0 (0)	0 (0)	0 (0)	14 (82.35)
Ebstein anomaly (*n* = 8)	1 (12.5)	3 (37.5)	0 (0)	0 (0)	0 (0)	4 (50)
Tricuspid stenosis (*n* = 1)	0 (0)	1 (100)	0 (0)	0 (0)	0 (0)	0 (0)
Pulmonary stenosis (*n* = 183)	34 (18.58)	9 (4.92)	0 (0)	0 (0)	0 (0)	140 (76.5)
Pulmonary valve dysplasia (*n* = 38)	8 (21.05)	0 (0)	0 (0)	0 (0)	0 (0)	30 (78.95)
Pulmonary atresia and intact ventricular septum (*n* = 33)	8 (24.24)	0 (0)	0 (0)	0 (0)	0 (0)	25 (75.75)
Pulmonary atresia and ventricular septal defect (*n* = 22)	5 (22.73)	4 (18.18)	0 (0)	0 (0)	0 (0)	13 (59.09)
Tetralogy of Fallot (*n* = 157)	11 (7)	41 (26.11)	0 (0)	1 (0.64)	0 (0)	104 (66.24)
Truncus arteriosus (*n* = 8)	0 (0)	0 (0)	1 (12.5)	0 (0)	0 (0)	7 (87.5)
Mitral valve abnormalities (*n* = 52)	10 (18.87)	4 (7.55)	0 (0)	0 (0)	0 (0)	38 (71.7)
Bicuspid aortic valve (*n* = 33)	4 (12.12)	2 (6.06)	0 (0)	0 (0)	0 (0)	27 (81.81)
Aortic valve stenosis (*n* = 52)	7 (13.46)	1 (1.92)	1 (1.92)	0 (0)	0 (0)	43 (82.7)
Aortic subvalvular stenosis (*n* = 4)	1 (25)	0 (0)	0 (0)	0 (0)	0 (0)	3 (75)
Coarctation of the aorta (*n* = 127)	28 (22.05)	15 (11.81)	0 (0)	0 (0)	0 (0)	83 (65.35)
Hypoplastic left heart syndrome (*n* = 17)	2 (11.76)	1 (5.88)	0 (0)	0 (0)	0 (0)	14 (82.35)
Transposition of the great arteries (*n* = 64)	10 (15.62)	6 (9.37)	1 (1.56)	1 (1.56)	0 (0)	46 (71.87)
Congenitally corrected transposition of the great arteries (*n* = 6)	0 (0)	0 (0)	0 (0)	0 (0)	0 (0)	6 (100)
Double outlet right ventricle (*n* = 44)	4 (9.09)	7 (15.9)	0 (0)	0 (0)	1 (2.27)	32 (72.72)
Double inlet left ventricle (*n* = 11)	1 (9.09)	1 (9.09)	0 (0)	0 (0)	0 (0)	9 (100)
Cor triatriatum (*n* = 3)	0 (0)	0 (0)	0 (0)	0 (0)	0 (0)	3 (100)
Abnormalities of atrial situs (*n* = 16)	0 (0)	1 (6.25)	0 (0)	0 (0)	0 (0)	14 (87.5)
Cardiomyopathies (*n* = 65)	3 (4.61)	0 (0)	0 (0)	0 (0)	0 (0)	62 (95.38)
Hypertrophic cardiomyopathy (*n* = 34)	2 (5.88)	0 (0)	0(0)	0 (0)	0 (0)	32 (94.11)
Dilated cardiomyopathy (*n* = 28)	1 (3.57)	0 (0)	0 (0)	0 (0)	0 (0)	27 (96.43)
Restrictive cardiomyopathy (*n* = 1)	0 (0)	0 (0)	0 (0)	0 (0)	0 (0)	1 (100)
Left ventricular non compaction (*n* = 2)	0 (0)	0 (0)	0 (0)	0 (0)	0 (0)	2 (100)
Rheumatic heart disease (*n* = 11)	1 (9.09)	0 (0)	0 (0)	0 (0)	0 (0)	10 (90.9)
Myocarditis (*n* = 50)	8 (16)	0 (0)	0 (0)	0 (0)	0 (0)	42 (84)
Pericardial disease (*n* = 71)	14 (19.71)	1 (1.41)	0 (0)	0 (0)	0 (0)	56 (78.87)
Acute pericarditis (*n* = 67)	13 (19.4)	1 (1.49)	0(0)	0 (0)	0 (0)	53 (79.1)
Chronic pericarditis (*n* = 4)	1 (25)	0 (0)	0 (0)	0 (0)	0 (0)	3 (75)
Kawasaki disease (*n* = 8)	1 (12.5)	0 (0)	0 (0)	0 (0)	0 (0)	7 (87.5)
Pulmonary hypertension (*n* = 25)	3 (12)	1 (4)	0 (0)	0 (0)	0 (0)	21 (84)
Cardiac tumours (*n* = 15)	1 (6.67)	0 (0)	0 (0)	0 (0)	0 (0)	14 (93.33)
Rhabdomyomas (*n* = 15)	1 (6.77)	0 (0)	0 (0)	0 (0)	0 (0)	14 (93.33)
cardiac rhythm disorders (*n* = 712)	91 (12.78)	21 (2.95)	0 (0)	1 (0.14)	0 (0)	599 (84.13)
Frequent supraventricular premature complexes (*n* = 48)	4 (8.33)	0 (0)	0 (0)	0 (0)	0 (0)	44 (91.67)
Frequent ventricular premature complexes (*n* = 78)	10 (12.82)	2 (2.56)	0 (0)	0 (0)	0 (0)	66 (84.61)
Ventricular pre-excitation (*n* = 130)	13 (10)	2 (1.53)	0 (0)	1 (0.8)	0 (0)	114 (87.69)
Atrial ectopic tachycardia (*n* = 130)	13 (10)	10 (7.69)	0 (0)	0 (0)	0 (0)	107 (82.3)
Atrioventricular re-entry tachycardia (*n* = 237)	29 (12.24)	6 (2.53)	0 (0)	0 (0)	0 (0)	202 (85.23)
Atrioventricular node re-entry tachycardia (*n* = 134)	26 (19.4)	6 (4.48)	0 (0)	0 (0)	0 (0)	102 (76.12)
Atrial flutter (*n* = 15)	1 (6.67)	0 (0)	0 (0)	0 (0)	0 (0)	14 (93.33)
Atrial fibrillation (*n* = 4)	0 (0)	0 (0)	0 (0)	0 (0)	0 (0)	4 (100)
Ventricular tachycardia (*n* = 7)	2 (28.57)	0 (0)	0 (0)	0 (0)	0 (0)	5 (71.43)
Cardiac channelopathies (*n* = 47)	9 (19.15)	1 (2.1)	0 (0)	0 (0)	0 (0)	37 (78.72)
Long QT syndrome (*n* = 27)	4 (14.81)	0 (0)	0 (0)	0 (0)	0 (0)	23 (85.18)
Brugada syndrome (*n* = 20)	5 (25)	1 (5)	0 (0)	0 (0)	0 (0)	14 (70)
No morpho-functional abnormalities (*n* = 620)	127 (20.48)	25 (4)	0 (0)	0 (0)	0 (0)	599 (96.61)

Abbreviations: CLBBB, complete left bundle branch block; CRBBB, complete right bundle branch block; IRBBB, incomplete right bundle branch block; LAFB, left anterior fascicular block.

## Data Availability

Data supporting the results of this article are available from the corresponding author upon reasonable request.

## Supplementary Materials

The following supporting information can be downloaded at: https://www.mdpi.com/article/10.3390/jcdd11040129/s1, Table S1: Prevalence and diagnostic accuracy of Incomplete right bundle branch block in paediatric heart disease; Table S2: Prevalence and diagnostic accuracy of complete right bundle branch block in paediatric heart disease; Table S3: Prevalence and diagnostic accuracy of complete left bundle branch block in paediatric heart disease; Table S4: Prevalence and diagnostic accuracy of left anterior fascicular block in paediatric heart disease; Table S5: Prevalence and diagnostic accuracy of complete right bundle branch block + left anterior fascicular block in paediatric heart disease.
