# Supplementary material for: Prevalence and Clinical Significance of Intraventricular Conduction Disturbances in Hospitalized Children
Source: J Cardiovasc Dev Dis. 2024 Apr 22;11(4):129. doi: 10.3390/jcdd11040129 (PMC11050792; doi:10.3390/jcdd11040129)
Supplement: Supplementary file 1 [file jcdd-11-00129-s001.zip › jcdd-2934559-supplementary.pdf]

Table S1. Prevalence and diagnostic accuracy of Incomplete right bundle branch block in paediatric heart disease.

	Sensitivity	Specificity	PPV	NPV	PA
Congenital heart diseases (n=2369)	19.1(17.6-20.8)	85.1(83.3-86.9)	65.3(62.0-68.4)	41.9(41.2-42.6)	46.0(44.4-47.5)
Patent foramen ovale (n=109)	8.3 (3.8-15.1)	82.4 (81.1-83.5)	1.3 (0.7-2.4)	96.9 (96.7-97.1)	80.3 (79.1-81.6)
Secundum atrial septal defect (n=847)	26.3 (23.3-29.4)	85.0 (83.7-86.3)	32.1 (26.1-35.1)	81.1 (80.4-81.7)	72.6 (71.1-73.9)
Primum atrial septal defect (n=11)	81.8 (48.2-97.7)	82.8 (81.5-83.9)	1.3 (0.8-1.7)	99.9 (99.7-99.9)	82.8 (81.5-83.9)
Atrioventricular septal defect (n=77)	10.4(4.6-19.4)	82.4(81.2-83.6)	1.1(0.6-2.2)	97.9(97.7-98.0)	81.0(79.8-82.2)
Ventricular septal defect (n=453)	12.8(9.9-16.2)	82.0(80.7-83.3)	8.3(6.6-10.5)	88.0(87.6-88.4)	74.1(72.8-75.5)
Patent ductus arteriosus (n=534)	15.3(12.4-18.7)	82.3(81.0-83.6)	11.8(9.8-14.2)	86.3(85.8-86.8)	73.3(71.9-74.7)
Aortopulmunary window (n=10)	20.0(2.5-55.6)	82.6(81.4-83.8)	0.3(0.1-1.0)	99.8(99.7-99.8)	82.5(81.2-83.6)
Abnormal origin of coronary artery from inappropriate situs (n=14)	21.4(4.7-50.8)	82.6(81.4-83.8)	0.4(0.2-1.2)	99.7(99.6-99.7)	82.4(81.2-83.6)
Anomalous left coronary artery from the pulmonary artery (n=2)	-	-	-	-	-
Anomalies of the aortic arch (n=61)	18.0(9.4-30.0)	82.6(81.4-83.8)	1.6(0.9-2.7)	98.5(98.3-98.6)	81.6(80.4-82.8)
Aberrant origin of the subclavian artery (n=9)	22.2(2.8-60.0)	82.6(81.4-83.8)	0.3(0.1-1.0)	99.8(99.7-99.8)	82.4(81.3-83.7)
Partially anomalous pulmonary venous connection (n=2)	-	-	-	-	-
Totally anomalous pulmonary venous connection (n=19)	15.8(3.4-39.6)	82.6(81.4-83.8)	0.4(0.1-1.2)	99.5(99.4-99.6)	82.3(81.1-83.5)
Tricuspid valve atresia (n=22)	4.5(0.1-22.8)	82.5(81.3-83.7)	0.1(0.0-1.0)	99.3(99.3-99.4)	82.1(80.9-83.3)
Tricuspid valve dysplasia (n=17)	5.9(0.1-28.7)	82.6(81.3-83.7)	0.1(0.0-1.0)	99.5(99.4-99.6)	82.2(81.0-83.4)
Ebstein anomaly (n=8)	12.5(0.3-52.6)	82.6(81.4-83.8)	0.1(0.0-0.9)	99.8(99.7-99.8)	82.5(81.2-83.6)
Tricuspid stenosis (n=1)	-	-	-	-	-
Pulmunary stenosis (n=183)	18.6(13.2-25.0)	82.7(81.4-83.9)	4.9(3.6-6.6)	95.5(95.2-95.8)	79.7(78.5-81.0)
Pulmunary valve dysplasia (n=38)	21.0(9.5-37.3)	82.6(81.4-83.8)	1.15(0.6-2.1)	99.1(98.9-99.2)	82.1(80.8-83.2)
Pulmunary atresia and intact ventriculum septum (n=33)	24.2(11.1-42.3)	82.7(81.5-83.8)	1.15(0.6-2.1)	99.2(99.1-99.3)	82.2(81.0-83.3)
Pulmunary atresia and ventricular septal defect (n=22)	22.7(7.8-45.3)	82.6(81.4-83.8)	0.7(0.3-1.5)	99.4(99.3-99.6)	82.3(81.1-83.5)
Tetralogy of Fallot (n=157)	7.0(3.5-12.2)	82.2(80.9-83.4)	1.6(0.9-2.8)	95.6(95.4-95.8)	79.2(77.9-80.5)
Truncus arteriosus (n=8)	-	-	-	-	-
Mitral valve abnormalities (n=53)	18.9(9.4-32.0)	82.6(81.4-83.8)	1.4(0.8-2.5)	98.7(98.5-98.8)	81.8(80.6-83.0)
Bicuspid aortic valve (n=33)	12.1(3.4-28.2)	82.6(81.4-83.7)	0.6(0.2-1.4)	99.1(99.0-99.2)	82.0(80.8-83.1)
Aortic valve stenosis (n=52)	13.5(5.6-25.8)	82.6(81.3-83.7)	1.0(0.5-2.0)	98.6(80.4-98.8)	81.7(80.4-82.9)
Aortic subvalvar stenosis (n=4)	25.0(0.6-80.6)	82.6(81.4-83.8)	0.1(0.0-0.7)	99.9(99.8-99.9)	82.6(81.3-83.7)
Coartaction of the aorta (n=127)	22.0(15.2-30.2)	82.8(81.5-83.9)	4.0(2.9-5.5)	97.0(96.7-97.2)	80.8(79.6-82.0)
Hypoplastic left heart syndrome (n=17)	11.8(1.5-36.4)	82.6(81.4-83.8)	0.3(0.1-1.0)	99.5(99.4-99.6)	82.3(81.1-83.5)
Transposition of the great arteries (n=64)	15.6(7.8-26.9)	82.6(81.4-83.8)	1.4(0.8-2.5)	98.3(98.2-98.5)	81.5(80.3-82.7)

Congenitally corrected transposition of the great arteries (n=6)	-	-	-	-	-
Double outlet right ventricle (n=44)	9.0(2.5-21.7)	82.5(81.3-83.7)	0.6(0.2-1.5)	98.8(98.7-98.9)	81.7(80.5-82.9)
Double inlet left ventricle (n=11)	0.2(0.0-1.3)	82.6(82.3-83.8)	0.1(0-1)	88.9(88.8-89)	74.9(73.6-76.2)
Cor triatratum (n=3)	-	-	-	-	-
Abnormalities of atrial situs (n=16)	-	-	-	-	-
Cardiomyopathies (n=65)	4.6(1.0-12.9)	82.4(81.2-83.6)	0.4(0.1-1.3)	98.1(98.0-98.2)	81.1(79.9-82.3)
Hypertrophic cardiomyopathy (n=34)	5.9(0.7-19.7)	82.5(81.3-83.7)	0.3(0.1-1.1)	99.0(98.9-99.1)	81.9(80.6-83)
Dilated cardiomyopathy (n=28)	3.6(0.1-18.3)	82.5(81.3-83.7)	0.1(0-1.0)	99.2(99.1-99.2)	82.0(80.7-83.1)
Restrictive cardiomyopathy (n=1)	-	-	-	-	-
Left ventricular non compaction (n=2)	-	-	-	-	-
Rheumatic heart disease (n=11)	9.1(0.2-41.3)	82.6(81.3-83.8)	0.1(0.0-0.9)	99.7(99.6-99.7)	82.4(81.2-83.6)
Myocarditis (n=50)	16.0(7.2-29.1)	82.6(81.4-83.8)	1.1(0.6-2.2)	98.7(98.6-98.9)	81.8(80.5-82.9)
Pericardial disease (n=71)	19.7(11.2-30.9)	82.7(81.4-83.8)	2.0(1.3-3.2)	98.3(98.1-98.5)	81.5(80.3-82.7)
Acute pericarditis (n=67)	19.4(10.8-30.9)	82.6(81.4-83.8)	1.9(1.1-3.0)	98.4(98.2-98.5)	81.6(80.4-82.8)
Chronic pericarditis (n=4)	25.0(0.6-80.6)	82.6(81.4-83.8)	0.1(0.0-0.8)	99.9(99.8-99.9)	82.6(81.4-83.7)
Kawasaki disease (n=8)	12.5(0.3-52.6)	82.6(81.4-83.8)	0.1(0.0-0.9)	99.8(99.7-99.8)	82.5(81.2-83.6)
Pulmonary hypertension (n=25)	12.0(2.5-31.2)	82.6(81.4-83.7)	0.4(0.1-1.2)	99.3(99.2-99.4)	82.1(80.9-83.32
Cardiac tumours (n=15)	6.7(0.2-31.9)	82.6(81.3-83.7)	0.12(0.0-0.9)	99.6(99.5-99.6)	82.3(81.1-83.5)
Rhabdomyomas (n=15)	6.7(0.2-31.9)	82.98(1.3-83.7)	0.12(0.0-0.9)	99.6(99.5-99.6)	82.3(81.1-83.5)
Disorder of the cardiac rhythm (n=712)	12.8(10.4-15.5)	81.6(80.2-82.9)	13.1(10.9-15.6)	81.2(80.7-81.7)	69.3(69.9-70.8)
Frequent supraventricular premature complexes (n=48)	8.3(2.3-20)	82.5(81.3-83.7)	0.6(0.2-1.5)	98.7(98.5-97.8)	81.6(80.4-82.8)
Frequent ventricular premature complexes (n=78)	12.8(6.3-22.3)	82.5(81.3-83.7)	1.4(0.82.5)	97.9(97.7-98.1)	81.1(79.9-82.4)
Ventricular pre-excitation (n=130)	10.0(5.4-16.5)	82.4(81.1-83.6)	1.9(1.1-3.1)	96.4(96.2-96.6)	80.0(78.7-81.2)
Atrial ectopic tachycardia (n=130)	10.0(5.4-16.5)	82.4(81.1-83.6)	1.9(1.1-3.1)	96.4(96.2-96.6)	80.0(78.7-81.2)
Atrioventricular reentry tachycardia (n=237)	12.2(8.3-17.1)	82.3(81.0-83.5)	4.2(3-5.8)	93.7(93.4-94.0)	78.1(76.8-79.4)
Atrioventricular node reentry tachycardia (n=134)	19.4(13.1-27.1)	81.3(80.1-82.6)	3.5(2.5-4.9)	96.7(96.4-96.9)	79.3(78.0-80.5)
Atrial flutter (n=15)	6.7(0.2-31.9)	82.6(81.4-83.7)	0.1(0.0-0.9)	99.6(99.5-99.6)	82.3(81.1-83.5)
Atrial fibrillation (n=4)	-	-	-	-	-
Ventricular tachycardia (n=7)	28.6(3.7-71.0)	82.6(81.4-83.8)	0.3(0.1-0.9)	99.85(99.8-99.9)	82.5(81.3-83.7)
Cardiac channelopathies (n=47)	19.1(9.1-33.3)	82.6(81.4-83.8)	1.3(0.7-2.3)	98.8(98.7-99.0)	81.9(80.7-83.1)
Long QT syndrome (n=27)	14.8(4.2-33.8)	82.6(81.4-83.8)	0.6(0.2-1.4)	99.3(99.2-99.4)	82.1(80.9-83.3)
Brugada syndrome (n=20)	25.0(8.7-49.1)	82.7(81.4-83.8)	0.7(0.3-1.5)	99.5(99.4-99.6)	82.4(81.1-83.5)
No morpho-functional abnormalities (n=620)	20.5(17.4-23.9)	67.7(65.4-69.9)	18.3(15.9-21.0)	70.6(69.6-71.7)	53.3(53.3-57.4)

Table S2. Prevalence and diagnostic accuracy of complete right bundle branch block in paediatric heart disease.

	Sensitivity	Specificity	PPV	NPV	PA
Congenital heart diseases (n=2369)	6.2(5.3-7.3)	97.3(96.4-98.1)	77.4(71.1-82.8)	41.6(41.2-41.9)	43.3(41.8-44.8)
Patent foramen ovale (n=109)	4.6(1.1-10.4)	95.2(94.4-95.9)	2.6(1.1-6.0)	97.2(97.1-97.4)	92.7(91.9-93.5)
Secundum atrial septal defect (n=847)	5.0(3.7-6.8)	93.9(92.9-94.9)	22.5(17.3-28.8)	74.1(73.7-74.4)	71.1(69.5-72.6)
Primum atrial septal defect (n=11)	18.2(2.3-51.8)	95.2(94.5-95.9)	1.0(0.3-3.6)	99.8(99.7-99.8)	95.0(94.3-95.7)
Atrioventricular septal defect (n=77)	9.1(3.7-17.8)	95.3(94.6-95.9)	3.7(1.8-7.2)	98.2(98.0-98.3)	93.6(92.8-94.4)
Ventricular septal defect (n=453)	6.2(4.1-8.8)	95.4(94.6-96.1)	14.7(10.4-20.2)	88.8(88.6-89.1)	85.3(84.1-86.4)
Patent ductus arteriosus (n=534)	1.9(0.9-3.4)	94.8(94.0-95.5)	5.2(2.9-9.4)	86.2(86.0-86.4)	82.3(81.1-83.5)
Aortopulmunary window (n=10)	10.0(0.2-44.5)	95.2(94.5-95.9)	0.5(0.1-3.3)	99.8(99.7-99.8)	95.0(94.3-95.7)
Abnormal origin of coronary artery from inappropriate situs (n=14)	7.1(0.2-33.9)	95.2(94.5-95.9)	0.5(0.1-3.4)	99.7(99.6-99.7)	94.9(94.2-95.6)
Anomalous left coronary artery from the pulmonary artery (n=2)	-	-	-	-	-
Anomalies of the aortic arch (n=61)	4.9(1-13.7)	95.2(94.5-95.9)	1.6(0.5-4.6)	98.5(98.4-98.6)	93.8(93-94.6)
Aberrant origin of the subclavian artery (n=9)	-	-	-	-	-
Partially anomalous pulmonary venous connection (n=2)	50.0(1.3-98.7)	95.2(94.3-95.9)	0.5(0.1-2.1)	99.97(99.8-99.9)	95.2(94.5-95.9)
Totally anomalous pulmonary venous connection (n=19)	10.5(1.3-33.1)	95.2(94.5-95.9)	1.0(0.3-3.8)	99.5(99.5-99.6)	94.8(94.1-95.5)
Tricuspid valve atresia (n=22)	-	-	-	-	-
Tricuspid valve dysplasia (n=17)	11.8(1.5-36.4)	95.2(94.5-95.9)	1.0(0.3-3.8)	99.6(99.5-99.7)	94.9(94.2-95.5)
Ebstein anomaly (n=8)	37.5(8.5-75.5)	95.3(94.6-95.9)	1.6(0.6-3.8)	99.9(99.8-99.9)	95.2(94.5-95.8)
Tricuspid stenosis (n=1)	100.0(2.5-100.0)	95.2(94.5-95.9)	0.5(0.5-0.6)	100.0	95.2(94.5-95.9)
Pulmunary stenosis (n=183)	4.9(2.3-9.1)	95.2(94.5-95.9)	4.7(2.5-8.7)	95.4(95.3-95.6)	91.1(90.0-91.9)
Pulmunary valve dysplasia (n=38)	-	-	-	-	-
Pulmunary atresia and intact ventriculum septum (n=33)	-	-	-	-	-
Pulmunary atresia and ventricular septal defect (n=22)	18.2(5.2-40.3)	95.3(94.6-95.9)	2.1(0.9-5)	99.5(99.4-99.6)	94.9(94.1-95.5)
Tetralogy of Fallot (n=157)	26.1(19.4-33.7)	96.1(95.4-96.7)	21.5(16.8-27.1)	96.9(96.7-97.2)	93.3(92.5-94.1))
Truncus arteriosus (n=8)	-	-	-	-	-
Mitral valve abnormalities (n=53)	7.5(2.1-18.2)	95.2(94.5-95.9)	2.1(0.8-5.2)	98.7(98.6-98.8)	94.0(93.3-94.8)
Bicuspid aortic valve (n=33)	6.1(0.7-20.2)	95.5(94.8-96.1)	1.0(0.3-3.9)	99.2(99.2-99.3)	94.7(94.0-95.4)
Aortic valve stenosis (n=52)	1.9(0-10.3)	95.3(94.6-95.9)	0.5(0.1-3.5)	98.7(98.6-98.7)	94.1(93.3-94.8)
Aortic subvalvar stenosis (n=4)	-	-	-	-	-
Coartaction of the aorta (n=127)	11.8(6.8-18.7)	95.4(94.7-96.1)	7.8(4.9-12.3)	97.0(96.9-97.2)	92.8(91.9-93.6)
Hypoplastic left heart syndrome (n=17)	5.9(0.1-28.7)	95.2(94.5-95.9)	0.5(0.1-3.4)	99.6(99.5-99.6)	94.8(94.1-95.5)
Transposition of the great arteries (n=64)	9.4(3.5-19.3)	95.3(94.6-95.9)	3.1(1.5-6.6)	98.5(98.3-98.6)	93.9(93.1-94.6)
Congenitally corrected transposition of the great arteries (n=6)	-	-	-	-	-

Double outlet right ventricle (n=44)	15.9(6.6-30.1)	95.3(94.6-96.0)	3.7(1.9-7.1)	99.0(98.9-99.1)	94.5(93.7-95.1)
Double inlet left ventricle (n=11)	9.1(0.2-41.3)	95.2(94.5-95.9)	0.5(0.1-3.3)	99.7(99.7-99.8)	95.0(94.3-95.6)
Cor triatratum (n=3)	-	-	-	-	-
Abnormalities of atrial situs (n=16)	6.2(0.2-30.2)	95.2(94.5-95.9)	0.5(0.1-3.4)	99.6(99.5-99.6)	94.9(94.1-95.5)
Cardiomyopathies (n=65)	-	-	-	-	-
Hypertrophic cardiomyopathy (n=34)	-	-	-	-	-
Dilated cardiomyopathy (n=28)	-	-	-	-	-
Restrictive cardiomyopathy (n=1)	-	-	-	-	-
Left ventricular non compaction (n=2)	-	-	-	-	-
Rheumatic heart disease (n=11)	-	-	-	-	-
Myocarditis (n=50)	-	-	-	-	-
Pericardial disease (n=71)	1.4(0.0-7.6)	95.2(94.4-95.8)	0.5(0.1-3.6)	98.2(98.1-98.2)	93.5(92.7-94.2)
Acute pericarditis (n=67)	1.5(0.0-8.0)	95.2(94.4-95.8)	0.5(0.1-3.6)	98.3(98.2-98.3)	93.6(92.8-94.3)
Chronic pericarditis (n=4)	-	-	-	-	-
Kawasaki disease (n=8)	-	-	-	-	-
Pulmonary hypertension (n=25)	4.0(0.1-20.3)	95.2(94.4-95.8)	0.5(0.1-3.5)	99.4(99.3-99.4)	94.6(93.8-95.3)
Cardiac tumours (n=15)	-	-	-	-	-
Rhabdomyomas (n=15)	-	-	-	-	-
Disorder of the cardiac rhythm (n=712)	2.9(1.8-4.5)	94.8(94.0-95.5)	11.0(7.3-16.2)	81.8(81.6-82.0)	78.4(77.1-79.7)
Frequent supraventricular premature complexes (n=48)	-	-	-	-	-
Frequent ventricular premature complexes (n=78)	2.6(0.3-8.9)	95.2(94.4-95.8)	1.0(0.3-4.0)	98.0(97.9-98.1)	93.4(92.5-94.1)
Ventricular pre-excitation (n=130)	1.5(0.2-5.4)	95.1(94.4-95.7)	1.0(0.3-4.0)	96.6(96.6-96.7)	92.0(91.2-92.9)
Atrial ectopic tachycardia (n=130)	7.7(3.7-13.7)	95.3(94.6-95.9)	5.2(2.9-9.2)	96.8(96.7-97.0)	92.4(91.6-93.2)
Atrioventricular reentry tachycardia (n=237)	2.5(0.9-5.4)	95.1(94.3-95.7)	3.1(1.4-6.7)	93.9(93.8-94.0)	89.6(88.6-90.5)
Atrioventricular node reentry tachycardia (n=134)	4.5(1.7-9.5)	95.2(94.5-95.9)	3.1(1.4-6.7)	96.6(96.5-96.7)	92.2(91.3-93.0)
Atrial flutter (n=15)	-	-	-	-	-
Atrial fibrillation (n=4)	-	-	-	-	-
Ventricular tachycardia (n=7)	-	-	-	-	-
Cardiac channelopathies (n=47)	2.1(0-11.1)	95.2(94.5-95.8)	0.5(0.1-3.5)	98.8(98.7-98.8)	94.1(93.3-94.8)
Long QT syndrome (n=27)	-	-	-	-	-
Brugada syndrome (n=20)	5.0(0.1-24.9)	95.2(94.5-95.9)	0.5(0.1-3.4)	99.5(99.4-99.5)	94.8(94.0-95.4)
No morpho-functional abnormalities (n=620)	4.0(2.6-5.9)	95.1(94.3-95.8)	13.1(9.1-18.5)	84.3(84.1-84.6)	81.0(79.7-82.1)

Table S3. Prevalence and diagnostic accuracy of complete left bundle branch block in paediatric heart disease.

	Sensitivity	Specificity	PPV	NPV	PA
Congenital heart diseases (n=2369)	0.1(0.0-0.4)	100.0(99.8-100)	100.0	40.7	40.7(39.2-42.3)
Patent foramen ovale (n=109)	-	-	-	-	-
Secundum atrial septal defect (n=847)	0.1(0.0-0.7)	99.9(99.8-100.0)	33.3(4.3-84.6)	78.8	78.8(77.5-80.0)
Primum atrial septal defect (n=11)	-	-	-	-	-
Atrioventricular septal defect (n=77)	-	-	-	-	-
Ventricular septal defect (n=453)	0.2(0.0-1.2)	99.9(99.8-100.0)	33.3(4.3-84.6)	88.7(88.6-88.7)	88.6(87.6-89.6)
Patent ductus arteriosus (n=534)	-	-	-	-	-
Aortopulmunary window (n=10)	-	-	-	-	-
Abnormal origin of coronary artery from inappropriate situs (n=14)	-	-	-	-	-
Anomalous left coronary artery from the pulmonary artery (n=2)	-	-	-	-	-
Anomalies of the aortic arch (n=61)	-	-	-	-	-
Aberrant origin of the subclavian artery (n=9)	-	-	-	-	-
Partially anomalous pulmonary venous connection (n=2)	-	-	-	-	-
Totally anomalous pulmonary venous connection (n=19)	-	-	-	-	-
Tricuspid valve atresia (n=22)	-	-	-	-	-
Tricuspid valve dysplasia (n=17)	-	-	-	-	-
Ebstein anomaly (n=8)	-	-	-	-	-
Tricuspid stenosis (n=1)	-	-	-	-	-
Pulmunary stenosis (n=183)	-	-	-	-	-
Pulmunary valve dysplasia (n=38)	-	-	-	-	-
Pulmunary atresia and intact ventriculum septum (n=33)	-	-	-	-	-
Pulmunary atresia and ventricular septal defect (n=22)	-	-	-	-	-
Tetralogy of Fallot (n=157)	-	-	-	-	-
Truncus arteriosus (n=8)	12.5(0.3-52.6)	99.9(99.8-100.0)	33.3(4.8-83.3)	99.8(99.8-99.9)	99.8(99.6-99.9)
Mitral valve abnormalities (n=53)	1.9(0.0-10.1)	99.9(99.8-100.0)	33.3(4.4-84.4)	98.7(98.6-98.7)	98.6(98.2-99.0)
Bicuspid aortic valve (n=33)	-	-	-	-	-
Aortic valve stenosis (n=52)	1.9(0.0-10.3)	99.9(99.8-100.0)	33.3(4.4-84.4)	98.7	98.7(98.3-99.0)
Aortic subvalvar stenosis (n=4)	-	-	-	-	-
Coartaction of the aorta (n=127)	-	-	-	-	-
Hypoplastic left heart syndrome (n=17)	-	-	-	-	-
Transposition of the great arteries (n=64)	1.6(0.0-8.4)	99.9(99.8-100.0)	33.3(4.4-84.5)	98.4(98.4-98.5)	98.4(97.7-98.7)
Congenitally corrected transposition of the great arteries (n=6)	-	-	-	-	-

Double outlet right ventricle (n=44)	-	-	-	-	-
Double inlet left ventricle (n=11)	-	-	-	-	-
Cor triatratum (n=3)	-	-	-	-	-
Abnormalities of atrial situs (n=16)	-	-	-	-	-
Cardiomyopathies (n=65)	-	-	-	-	-
Hypertrophic cardiomyopathy (n=34)	-	-	-	-	-
Dilated cardiomyopathy (n=28)	-	-	-	-	-
Restrictive cardiomyopathy (n=1)	-	-	-	-	-
Left ventricular non compaction (n=2)	-	-	-	-	-
Rheumatic heart disease (n=11)	-	-	-	-	-
Myocarditis (n=50)	-	-	-	-	-
Pericardial disease (n=71)	-	-	-	-	-
Acute pericarditis (n=67)	-	-	-	-	-
Chronic pericarditis (n=4)	-	-	-	-	-
Kawasaki disease (n=8)	-	-	-	-	-
Pulmonary hypertension (n=25)	-	-	-	-	-
Cardiac tumours (n=15)	-	-	-	-	-
Rhabdomyomas (n=15)	-	-	-	-	-
Disorder of the cardiac rhythm (n=712)	-	-	-	-	-
Frequent supraventricular premature complexes (n=48)	-	-	-	-	-
Frequent ventricular premature complexes (n=78)	-	-	-	-	-
Ventricular pre-excitation (n=130)	-	-	-	-	-
Atrial ectopic tachycardia (n=130)	-	-	-	-	-
Atrioventricular reentry tachycardia (n=237)	-	-	-	-	-
Atrioventricular node reentry tachycardia (n=134)	-	-	-	-	-
Atrial flutter (n=15)	-	-	-	-	-
Atrial fibrillation (n=4)	-	-	-	-	-
Ventricular tachycardia (n=7)	-	-	-	-	-
Cardiac channelopathies (n=47)	-	-	-	-	-
Long QT syndrome (n=27)	-	-	-	-	-
Brugada syndrome (n=20)	-	-	-	-	-
No morpho-functional abnormalities (n=620)	-	-	-	-	-

Table S4 Prevalence and diagnostic accuracy of left anterior fascicular block in paediatric heart disease.-

	Sensitivity	Speci-ficity	PPV	NPV	PA
Congenital heart diseases (n=2369)	0.2(0.1-0.5)	99.9(99.7-100.0)	83.3(36.9-97.7)	40.7(40.6-40.7)	40.8(39.2-42.3)
Patent foramen ovale (n=109)	-	-	-	-	-
Secundum atrial septal defect (n=847)	0.1(0.0-0.7)	99.8(99.6-99.9)	16.7(2.3-63.1)	78.8(78.7-78.8)	78.7(77.4-79.9)
Primum atrial septal defect (n=11)	-	-	-	-	-
Atrioventricular septal defect (n=77)	2.6(0.3-9.1)	99.9(99.8-100.0)	33.3(98.0-98.2)	98.1(98.0-98.2)	98.0(97.5-98.4)
Ventricular septal defect (n=453)	0.2(0.0-1.2)	99.8(99.7-99.9)	16.7(2.363.1)	88.7(88.6-88.7)	88.5(87.5-89.5)
Patent ductus arteriosus (n=534)	-	-	-	-	-
Aortopulmunary window (n=10)	-	-	-	-	-
Abnormal origin of coronary artery from inappropriate situs (n=14)	-	-	-	-	-
Anomalous left coronary artery from the pulmonary artery (n=2)	-	-	-	-	-
Anomalies of the aortic arch (n=61)	-	-	-	-	-
Aberrant origin of the subclavian artery (n=9)	-	-	-	-	-
Partially anomalous pulmonary venous connection (n=2)	-	-	-	-	-
Totally anomalous pulmonary venous connection (n=19)	-	-	-	-	-
Tricuspid valve atresia (n=22)	4.5(0.1-22.9)	99.9(99.7-100.0)	16.7(2.4-62.2)	99.5(99.4-99.5)	99.3(99.0-99.6)
Tricuspid valve dysplasia (n=17)	-	-	-	-	-
Ebstein anomaly (n=8)	-	-	-	-	-
Tricuspid stenosis (n=1)	-	-	-	-	-
Pulmunary stenosis (n=183)	-	-	-	-	-
Pulmunary valve dysplasia (n=38)	-	-	-	-	-
Pulmunary atresia and intact ventriculum septum (n=33)	-	-	-	-	-
Pulmunary atresia and ventricular septal defect (n=22)	-	-	-	-	-
Tetralogy of Fallot (n=157)	0.6(0.0-3.5)	99.9(99.7-99.9)	16.7(2.3-63.0)	96.1(96.0-96.1)	96.0(95.3-96.6)
Truncus arteriosus (n=8)	-	-	-	-	-
Mitral valve abnormalities (n=53)	-	-	-	-	-
Bicuspid aortic valve (n=33)	-	-	-	-	-
Aortic valve stenosis (n=52)	-	-	-	-	-
Aortic subvalvar stenosis (n=4)	-	-	-	-	-
Coartaction of the aorta (n=127)	-	-	-	-	-
Hypoplastic left heart syndrome (n=17)	-	-	-	-	-
Transposition of the great arteries (n=64)	1.6(0.0-8.4)	99.9(99.7-100.0)	16.7(2.3-62.8)	98.4(98.4-98.5)	98.3(97.8-98.7)
Congenitally corrected transposition of the great arteries (n=6)	-	-	-	-	-

Double outlet right ventricle (n=44)	-	-	-	-	-
Double inlet left ventricle (n=11)	-	-	-	-	-
Cor triatratum (n=3)	-	-	-	-	-
Abnormalities of atrial situs (n=16)	-	-	-	-	-
Cardiomyopathies (n=65)	-	-	-	-	-
Hypertrophic cardiomyopathy (n=34)	-	-	-	-	-
Dilated cardiomyopathy (n=28)	-	-	-	-	-
Restrictive cardiomyopathy (n=1)	-	-	-	-	-
Left ventricular non compaction (n=2)	-	-	-	-	-
Rheumatic heart disease (n=11)	-	-	-	-	-
Myocarditis (n=50)	-	-	-	-	-
Pericardial disease (n=71)	-	-	-	-	-
Acute pericarditis (n=67)	-	-	-	-	-
Chronic pericarditis (n=4)	-	-	-	-	-
Kawasaki disease (n=8)	-	-	-	-	-
Pulmonary hypertension (n=25)	-	-	-	-	-
Cardiac tumours (n=15)	-	-	-	-	-
Rhabdomyomas (n=15)	-	-	-	-	-
Disorder of the cardiac rhythm (n=712)	0.1(0.0-0.8)	99.8(99.6-99.9)	16.7(2.3-63.1)	82.2(82.1-82.2)	82.1(80.9-83.2)
Frequent supraventricular premature complexes (n=48)	-	-	-	-	-
Frequent ventricular premature complexes (n=78)	-	-	-	-	-
Ventricular pre-excitation (n=130)	0.8(0.0-4.2)	99.9(99.7-100.0)	16.7(2.3-63.0)	96.8(96.7-96.8)	96.6(96-97.2)
Atrial ectopic tachycardia (n=130)	-	-	-	-	-
Atrioventricular reentry tachycardia (n=237)	-	-	-	-	-
Atrioventricular node reentry tachycardia (n=134)	-	-	-	-	-
Atrial flutter (n=15)	-	-	-	-	-
Atrial fibrillation (n=4)	-	-	-	-	-
Ventricular tachycardia (n=7)	-	-	-	-	-
Cardiac channelopathies (n=47)	-	-	-	-	-
Long QT syndrome (n=27)	-	-	-	-	-
Brugada syndrome (n=20)	-	-	-	-	-
No morpho-functional abnormalities (n=620)	-	-	-	-	-

Table S5. Prevalence and diagnostic accuracy of complete right bundle branch block + left anterior fascicular block in paediatric heart disease.

	Sensitivity	Specificity	PPV	NPV	PA
Congenital heart diseases (n=2369)	0.2(0.1-0.5)	100.0(99.8-100.0)	100.0	40.7(40.7-40.8)	40.8(39.3-42.4)
Patent foramen ovale (n=109)	-	-	-	-	-
Secundum atrial septal defect (n=847)	-	-	-	-	-
Primum atrial septal defect (n=11)	-	-	-	-	-
Atrioventricular septal defect (n=77)	6.5(2.1-14.5)	99.97(99.7-100.0)	83.3(22.8-77.2)	98.2(98.1-98.3)	98.2(97.6-98.5)
Ventricular septal defect (n=453)	-	-	-	-	-
Patent ductus arteriosus (n=534)	-	-	-	-	-
Aortopulmunary window (n=10)	-	-	-	-	-
Abnormal origin of coronary artery from inappropriate situs (n=14)	-	-	-	-	-
Anomalous left coronary artery from the pulmonary artery (n=2)	-	-	-	-	-
Anomalies of the aortic arch (n=61)	-	-	-	-	-
Aberrant origin of the subclavian artery (n=9)	-	-	-	-	-
Partially anomalous pulmonary venous connection (n=2)	-	-	-	-	-
Totally anomalous pulmonary venous connection (n=19)	-	-	-	-	-
Tricuspid valve atresia (n=22)	-	-	-	-	-
Tricuspid valve dysplasia (n=17)	-	-	-	-	-
Ebstein anomaly (n=8)	-	-	-	-	-
Tricuspid stenosis (n=1)	-	-	-	-	-
Pulmunary stenosis (n=183)	-	-	-	-	-
Pulmunary valve dysplasia (n=38)	-	-	-	-	-
Pulmunary atresia and intact ventriculum septum (n=33)	-	-	-	-	-
Pulmunary atresia and ventricular septal defect (n=22)	-	-	-	-	-
Tetralogy of Fallot (n=157)	-	-	-	-	-
Truncus arteriosus (n=8)	-	-	-	-	-
Mitral valve abnormalities (n=53)	-	-	-	-	-
Bicuspid aortic valve (n=33)	-	-	-	-	-
Aortic valve stenosis (n=52)	-	-	-	-	-
Aortic subvalvar stenosis (n=4)	-	-	-	-	-
Coartaction of the aorta (n=127)	-	-	-	-	-
Hypoplastic left heart syndrome (n=17)	-	-	-	-	-
Transposition of the great arteries (n=64)	-	-	-	-	-
Congenitally corrected transposition of the great arteries (n=6)	-	-	-	-	-

Double outlet right ventricle (n=44)	2.3(0.0-12.0)	99.9(99.7-100.0)	16.7(2.3-99.0)	98.9(98.9-99.0)	98.8(98.4-99.1)
Double inlet left ventricle (n=11)	-	-	-	-	-
Cor triatratum (n=3)	-	-	-	-	-
Abnormalities of atrial situs (n=16)	-	-	-	-	-
Cardiomyopathies (n=65)	-	-		-	-
Hypertrophic cardiomyopathy (n=34)	-	-	-	-	-
Dilated cardiomyopathy (n=28)	-	-	-	-	-
Restrictive cardiomyopathy (n=1)	-	-	-	-	-
Left ventricular non compaction (n=2)	-	-	-	-	-
Rheumatic heart disease (n=11)	-	-	-	-	-
Myocarditis (n=50)	-	-	-	-	-
Pericardial disease (n=71)	-	-	-	-	-
Acute pericarditis (n=67)	-	-	-	-	-
Chronic pericarditis (n=4)	-	-	-	-	-
Kawasaki disease (n=8)	-	-	-	-	-
Pulmonary hypertension (n=25)	-	-	-	-	-
Cardiac tumours (n=15)	-	-	-	-	-
Rhabdomyomas (n=15)	-	-	-	-	-
Disorder of the cardiac rhythm (n=712)	-	-	-	-	-
Frequent supraventricular premature complexes (n=48)	-	-	-	-	-
Frequent ventricular premature complexes (n=78)	-	-	-	-	-
Ventricular pre-excitation (n=130)	-	-	-	-	-
Atrial ectopic tachycardia (n=130)	-	-	-	-	-
Atrioventricular reentry tachycardia (n=237)	-	-	-	-	-
Atrioventricular node reentry tachycardia (n=134)	-	-	-	-	-
Atrial flutter (n=15)	-	-	-	-	-
Atrial fibrillation (n=4)	-	-	-	-	-
Ventricular tachycardia (n=7)	-	-	-	-	-
Cardiac channelopathies (n=47)	-	-	-	-	-
Long QT syndrome (n=27)	-	-	-	-	
Brugada syndrome (n=20)	-	-	-	-	
No morpho-functional abnormalities (n=620)			-		
